# New‐Onset Device‐Detected Atrial Fibrillation in Patients With Atrial Floating Dipole Implantable Cardioverter‐Defibrillators: A Propensity Score‐Matched Comparison With Conventional Dual‐Chamber Systems

**DOI:** 10.1111/jce.16666

**Published:** 2025-04-02

**Authors:** Gianfranco Mitacchione, Antonio Curnis, Eduardo Celentano, Giovanni Rovaris, Antonella Battista, Massimiliano Marini, Paolo Della Bella, Vincenzo Ezio Santobuono, Mauro Biffi, Luca Tomasi, Matteo Baroni, Luca Bontempi, Gerardo Nigro, Emilio Di Lorenzo, Donatella Ruggiero, Fabio Franculli, Patrizia Pepi, Miguel Viscusi, Davide Saporito, Matteo Bertini, Gaetano Senatore, Stefano Pedretti, Domenico Pecora, Giovanni Battista Forleo, Francesco Solimene, Valerio Giordano, Riccardo Sacchi, Daniele Giacopelli, Alessio Gargaro, Fabrizio Caravati

**Affiliations:** ^1^ ASST Spedali Civili, Department of Medical and Surgical Specialties, Radiological Sciences and Public Health University of Brescia Brescia Italy; ^2^ Humanitas Gavazzeni Bergamo Italy; ^3^ Fondazione IRCCS San Gerardo dei Tintori Monza Italy; ^4^ Arcispedale Santa Maria Nuova Reggio Emilia Italy; ^5^ Ospedale Santa Chiara Trento Italy; ^6^ Ospedale San Raffaele Milano Italy; ^7^ Policlinico Universitario di Bari Bari Italy; ^8^ Policlinico Sant'Orsola‐Malpighi Bologna Italy; ^9^ Azienda Ospedaliera Universitaria Integrata Verona Verona Italy; ^10^ ASST Grande Ospedale Metropolitano Niguarda Milano Italy; ^11^ Ospedale di Seriate Seriate Italy; ^12^ Università ‘Vanvitelli’, Ospedale Monaldi Napoli Italy; ^13^ Ospedale Monaldi Napoli Italy; ^14^ Ospedale Pio XI Desio Italy; ^15^ Ospedale San Giovanni di Dio e Ruggi d'Aragona Salerno Italy; ^16^ Ospedale Carlo Poma Mantova Italy; ^17^ Ospedale di Caserta Caserta Italy; ^18^ Ospedale Infermi Rimini Italy; ^19^ Nuovo Arcispedale S.Anna Ferrara Italy; ^20^ Ospedale di Ciriè Ciriè Italy; ^21^ Ospedale San Paolo Milano Italy; ^22^ Fondazione Poliambulanza Istituto Ospedaliero Brescia Italy; ^23^ Ospedale Sacco Milano Italy; ^24^ Clinica Montevergine Mercogliano Italy; ^25^ Presidio Ospedaliero San Luca Vallo della Lucania Italy; ^26^ ASST Brianza ‐ Ospedale di Vimercate Vimercate Italy; ^27^ Biotronik Italia S.p.a. Cologno Monzese Italy; ^28^ ASST dei sette laghi Ospedale di Circolo Varese Italy

**Keywords:** device‐detected atrial fibrillation, DX ICD, floating atrial dipole, implantable cardioverter‐defibrillator, remote monitoring

## Abstract

**Background:**

Device‐detected subclinical atrial fibrillation (DDAF) is a significant risk factor for major cardiovascular events, especially in implantable cardioverter‐defibrillator (ICD) recipients. The DX ICD, which utilizes a single ventricular lead with a floating atrial dipole, has demonstrated superior performance in diagnosing DDAF compared to conventional single‐lead ICDs. However, comparisons between DX and dual‐chamber (DDD) ICDs for atrial monitoring are limited.

**Objective:**

To compare the incidence of newly detected DDAF in patients without an indication for atrial pacing who received either a DX or a standard DDD ICD.

**Methods:**

Remote transmissions from the Italian Home Monitoring Expert Alliance dataset were analyzed. DDAF incidence for different burden cutoffs ( ≥ 15 min, ≥ 6 h, and ≥ 24 h) was compared between groups using propensity score (PS) matching to adjust for baseline characteristics.

**Results:**

In a cohort of 1329 patients (527 with DX ICD and 802 with DDD ICD), 30.7% experienced DDAF lasting ≥ 15 min, 22.3% ≥ 6 h, and 14.0% ≥ 24 h during a median follow‐up of 4.5 years. DDAF incidence was lower in the DX ICD group for all burden cutoffs (*p* < 0.0001). However, after PS matching, DDAF rates were similar between groups, with no significant differences (*p* ≥ 0.36). Multivariate analysis identified age and 1‐month right ventricular pacing percentage as predictors of DDAF across all burden cutoffs, with no effect based on device type or programmed basic rate.

**Conclusion:**

In patients without atrial pacing indication or history of clinical atrial fibrillation at implantation, the DX ICD demonstrated DDAF detection capabilities comparable to DDD ICDs in a real‐world setting.

**Abbreviations**
AF: atrial fibrillation
AV: atrioventricular
DDAF: device‐detected subclinical AF
DDD ICD: dual‐chamber ICD
DX ICD: ICD with single ventricular lead and floating atrial dipole
ICD: implantable cardioverter‐defibrillator
PS: propensity score
RVP%: right ventricular pacing percentage

## 1 Introduction

Single‐chamber implantable cardioverter‐defibrillators (ICDs) are currently recommended over dual‐chamber (DDD) ICDs for primary prevention of sudden cardiac death in patients without an indication for atrial or atrioventricular (AV) sequential pacing [1]. This recommendation is based not on the potential disadvantages of atrial pacing compared to ventricular backup pacing [2], but rather on the lower risk of device‐related complications when an atrial lead is not implanted [3, 4]. Nevertheless, atrial lead implantation remains common even in patients who do not require pacing [5].

This practice may be partially justified by the increasingly recognized role of automated continuous atrial rhythm monitoring in the diagnosis of device‐detected subclinical atrial fibrillation (DDAF). Patients experiencing DDAF episodes are at a high risk of developing clinical atrial fibrillation (AF) [6]. Therefore, DDAF detection could be crucial for guiding the timely initiation of anticoagulation therapy and subsequent follow‐up [7].

In this context, implanting a single ventricular ICD lead with a floating atrial dipole (DX ICD, Biotronik SE & Co. KG, Berlin, Germany) in place of a DDD ICD in patients without an indication for atrial pacing may be an appealing option for long‐term atrial rhythm monitoring without increasing the number of leads of the ICD system [8, 9].

The objective of this analysis was to compare the incidence of new‐onset DDAF in patients without an indication for atrial pacing who received either a DX ICD or a standard DDD ICD in a real‐world setting. Additionally, we investigated the effect of atrial pacing on DDAF by comparing different pacing modes in dual‐chamber systems.

## 2 Methods

### 2.1 Study Design

This analysis was conducted within the framework of the Home Monitoring Expert Alliance (HMEA), an independent, ongoing project designed to test scientific hypotheses using a large, real‐world dataset generated by remote monitoring of cardiac implantable electronic devices [10]. Data are transmitted daily using the Home Monitoring System (Biotronik), prospectively collected, and pooled from networked clinics. The HMEA project has received approval from ethics committees, and all patients provided written consent for remote monitoring activation and data processing.

### 2.2 Sample Selection and Study Groups

From the HMEA database, we screened all patients with sinus rhythm who underwent successful implantation of a de novo DX ICD or a standard DDD ICD, with at least 1 month of follow‐up via remote transmissions. Patients were further selected by excluding those who, at the time of implantation, had the following characteristics: (i) a history of AF, (ii) a diagnosis of sinus node dysfunction with an indication for atrial pacing, or (iii) activation of a dual‐chamber rate‐responsive pacing mode.

Within this cohort, two groups were identified. The first group included patients implanted with a DX ICD system, which uses a single right ventricular screw‐in lead incorporating a floating atrial dipole for sensing, capable of providing complete dual‐chamber diagnostics [11, 12]. In this group, all patients received a defibrillation lead with the atrial dipole located 15 cm from the distal tip. The second group consisted of patients implanted with a standard DDD ICD with right atrial and right ventricular endocardial leads and dual‐chamber pacing programming (DDD). In the latter group, further subgroups were identified based on programmed basic rate: DDD with a basic rate of < 60 beats per minute (bpm) and DDD with a basic rate of ≥ 60 bpm.

### 2.3 Atrial Sensitivity Programming

In both groups, atrial sensitivity was set to default programming, utilizing automatic adaptation based on sensed amplitude with progressive decay toward a maximum sensitivity of 0.2 mV. In addition to the common programming of this dynamic sensing threshold, DX ICD devices incorporate a modified atrial input stage with specific filters for the floating atrial dipole and enhanced signal amplification, allowing up to four‐fold amplification of the atrial signal [13].

### 2.4 Study Endpoints

For each patient, we retrieved remote monitoring data on the DDAF burden, defined as the total time spent in DDAF within a single day. DDAF was identified based on an automatic device detection rate of 200 bpm in most cases. The study endpoints were the time to the first DDAF using three cutoffs for 24‐h DDAF burden: ≥ 15 min (1% of 24 h, the maximum resolution of daily remote transmissions), ≥ 6 h, and ≥ 24 h.

### 2.5 Statistical Analysis

Continuous variables are reported as medians with interquartile ranges (IQRs), while binary variables are presented as counts and percentages of non‐missing values. Differences between groups were assessed using the Mann–Whitney *U* test for continuous variables and the *χ* ^2^ or Fisher's exact test for binary/categorical variables.

To address the heterogeneity between the DX and DDD ICD groups, a subset of patients with standard DDD ICDs was selected using propensity score (PS) matching. The covariates included in the PS calculation were those that exhibited significant differences between the unmatched cohorts: age, CHA_2_DS_2_‐VASc score, secondary prevention indication, congenital cardiomyopathy, chronic heart failure, diabetes, and the 1‐month right ventricular pacing percentage (RVP%). After confirming satisfactory common support between groups (Figure S1), a PS‐based 1:1 match was performed using the nearest‐neighbor method with replacement. The adequacy of the common support between unmatched groups and the reduction in the absolute standardized mean differences between matched groups for all baseline variables were verified (Figure S2).

Kaplan–Meier curves for DDAF‐free rates were generated for all burden cutoffs and compared between unmatched and matched groups using the univariable Cox regression test. Potential predictors of DDAF were analyzed using multivariable Cox proportional hazards regression models, including the type of implanted device, age, sex, programmed basic rate, and 1‐month RVP%. Results were reported as hazard ratios with corresponding 95% confidence intervals. Comparisons of DDAF‐free rates according to different burden cutoffs were also performed by basic‐rate subgroups ( < 60 bpm vs. ≥ 60 bpm) within DDD ICD systems.

*p* values < 0.05 were considered statistically significant. All statistical analyses were conducted using Stata 18.0MP (StataCorp LLC, College Station, Texas).

## 3 Results

### 3.1 Study Population

**Figure 1:**
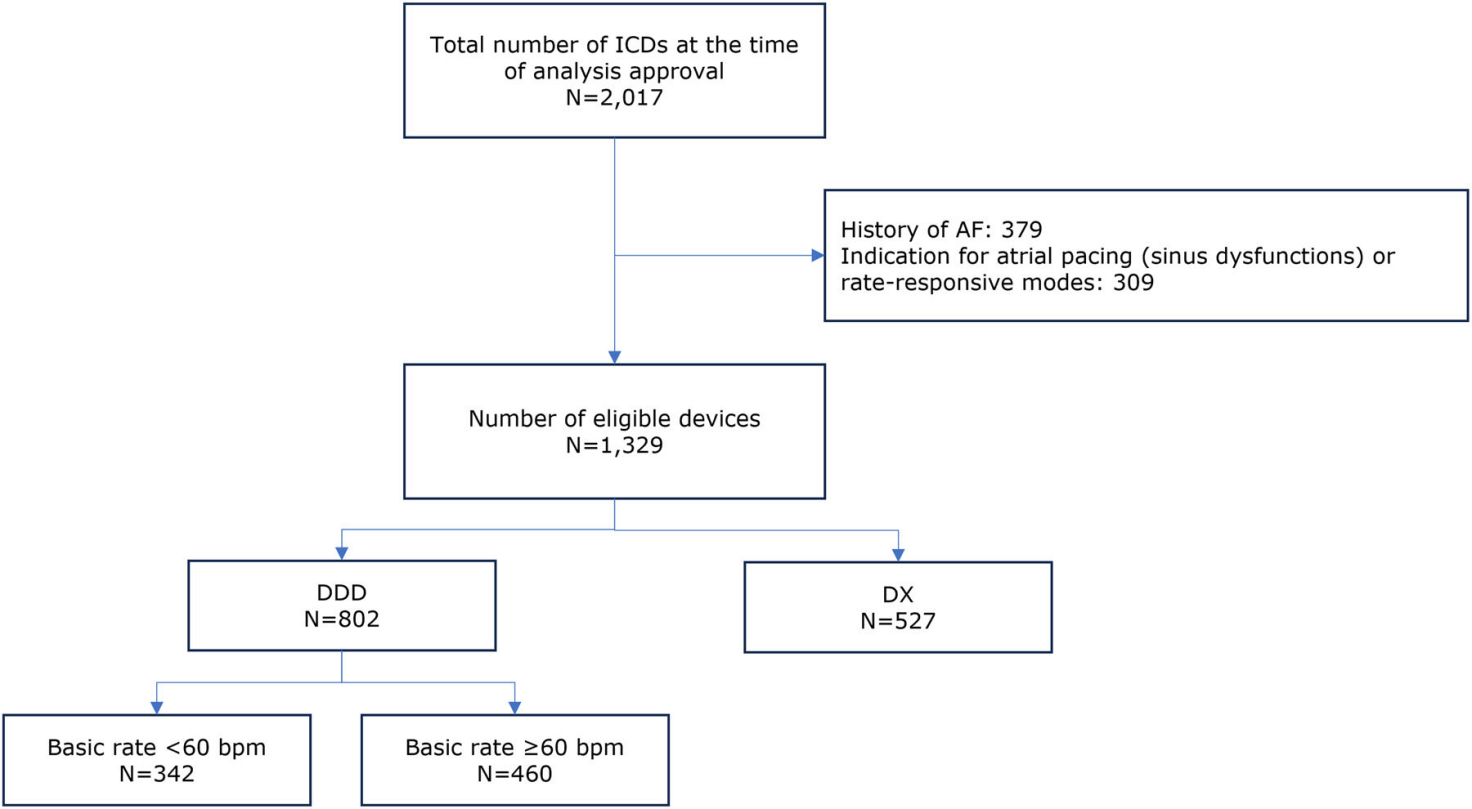
Flowchart of selection of the study population. Abbreviations: AF = atrial fibrillation; bpm = beats per minute; ICD = implantable cardioverter‐defibrillator.

Out of the 2017 ICDs with atrial sensing capabilities currently recorded in the HMEA database, 379 patients (18.8%) were excluded due to a history of clinical AF, and 309 patients (15.3%) were excluded because of an indication for atrial pacing related to sinus node dysfunction or the activation of a rate‐responsive pacing mode. The remaining 1329 patients were included in the analysis: 527 received a DX ICD and 802 received a standard DDD ICD device (Figure 1).

Baseline patient characteristics are provided in Table 1. When compared with DDD ICD, patients implanted with a DX ICD were younger (median age 63 vs. 67 years, *p* < 0.001) and had a lower New York Heart Association functional class (*p* < 0.001), a lower CHA_2_DS_2_‐VASc score (*p* < 0.001), and a lower prevalence of diabetes (18.8% vs. 24.6%, *p* = 0.026). A higher proportion of patients implanted with secondary prevention indication (31.3% vs. 24.1%, *p* = 0.009) and congenital cardiomyopathy (13.3% vs. 7.6%, *p* = 0.002) were also found in the DX ICD group.

**Table 1:** Baseline characteristics in the overall population and according to study groups.

	All patients (*n* = 1329)	DX ICD (a) (*n* = 527)	DDD ICD (b) (*n* = 802)	DDD < 60 bpm subgroup (c) (*n* = 342)	DDD ≥ 60 bpm subgroup (d) (*n* = 460)	*p* value (a vs. b)	*p* value (c vs. d)
Age (years)	66 [56–74]	63 [53–71]	67 [58–75]	66 [56–74]	68 [59–76]	< 0.001	< 0.001
Sex (female)	214 (16.1%)	79 (15.0%)	135 (16.9%)	55 (16.1%)	80 (17.4%)	0.37	0.63
NYHA Class						< 0.001	0.78
I–II	624 (79.7%)	232 (80.3%)	392 (79.3%)	148 (77.0%)	244 (80.8%)		
III–IV	159 (20.3%)	57 (19.7%)	102 (20.7%)	44 (23.0%)	58 (19.2%)		
LVEF (%)	31 [29–37]	32 [28–40]	30 [30–35]	30 [30–38]	30 [30–35]	0.51	0.48
CHA_2_DS_2_VASc score	2 [1–4]	2 [1–3]	2 [1–4]	2 [1–3]	3 [1–4]	< 0.001	< 0.001
Secondary prevention ICD indication	293 (27.1%)	138 (31.3%)	155 (24.1%)	74 (28.7%)	81 (21.1%)	0.009	0.028
Ischemic CMP	672 (59.1%)	261 (56.9%)	411 (60.6%)	172 (60.6%)	239 (60.7%)	0.206	0.98
Nonischemic CMP	274 (24.9%)	100 (22.5%)	174 (26.5%)	65 (24.3%)	109 (27.9%)	0.131	0.30
Congenital CMP	109 (9.9%)	59 (13.3%)	50 (7.6%)	26 (9.85%)	24 (6.14%)	0.002	0.08
Diabetes	243 (22.2%)	84 (18.8%)	159 (24.6%)	58 (22%)	101 (26.4%)	0.026	0.20
CKD	116 (10.5%)	47 (10.5%)	69 (10.5%)	27 (9.96%)	42 (10.9%)	0.99	0.69
Stroke/TIA	101 (9.2%)	32 (7.14%)	69 (10.6%)	10 (3.7%)	59 (15.4%)	0.053	< 0.001
AV Block	31 (2.8%)	14 (3.1%)	17 (2.6%)	8 (3.0%)	9 (2.3%)	0.63	0.58
Therapy							
β‐blockers	848 (77.9%)	351 (79.4%)	497 (76.8%)	210 (78.1%)	287 (75.9%)	0.31	0.52
Diuretics	670 (61.9%)	257 (58.9%)	413 (63.9%)	163 (61%)	250 (66%)	0.10	0.20
ACE inhibitors	575 (53.3%)	232 (53.2%)	343 (53.3%)	156 (59.1%)	187 (49.3%)	0.97	0.015
CCB	133 (12.9%)	55 (13.2%)	78 (12.7%)	34 (13.9%)	44 (11.9%)	0.83	0.46
ARB	107 (10.4%)	47 (11.2%)	60 (9.8%)	24 (9.9%)	36 (9.8%)	0.47	0.99
Amiodarone	111 (10.4%)	48 (11.2%)	63 (9.9%)	28 (10.6%)	35 (9.4%)	0.51	0.62
Pacing mode							
DDD	802 (60.3%)	—	802 (100%)	342 (100%)	460 (100%)	—	—
VDD	64 (4.8%)	64 (12.1%)*	0 (0%)	0 (0%)	0 (0%)	—	—
VVI	463 (34.8%)	463 (87.9%)*	0 (0%)	0 (0%)	0 (0%)	—	—
Basic rate (bpm)	50 [40–60]	40 [40–40]	60 [50–60]	—	—	< 0.001	—
AV delay (ms)	160 [140–200]	200 [140–240]**	160 [140–190]	160 [140–200]	160 [140–185]	< 0.001	0.052
AV hysteresis	649 (48.8%)	44 (8.4%)	605 (75.4%)	231 (67.5%)	374 (81.3%)	< 0.001	< 0.001
1‐month AP (%)	3.7 [0.1–23.3]	—	3.7 [0.1–23.3]	0.2 [0.0–3.7]	13.1 [2.2–40.7]	—	< 0.001
1‐month RVP (%)	0.3 [0.0–2.0]	0.0 [0.0–0.1]	1.0 [0.1–3.8]	0.8 [0.0–3.0]	1.2 [0.3–4.9]	< 0.001	0.44
Atrial sensing amplitude at 1 month (mV)	3.92 [2.76–5.23]	4.58 [3.10–5.87]	3.66 [2.59–4.72]	—	—	< 0.001	—
Atrial sensing amplitude at study termination (mV)	3.65 [2.35–5.05]	4.29 [2.63–5.98]	3.38 [2.31–4.59]	—	—	< 0.001	—

*Note:* Data are median [interquartile range] or count (%).

Abbreviations: ACE = angiotensin‐converting enzyme, AP = atrial pacing, ARB = angiotensin receptor blocker, AV = atrioventricular, bpm = beats per minute, CCB = calcium channel blocker, CKD = chronic kidney disease, CMP = cardiomyopathy, ICD = implantable cardioverter‐defibrillator, LVEF = left ventricular ejection fraction, NYHA = New York Heart Association, RVP = right ventricular pacing, TIA = transient ischemic attack.

* DX ICD systems provide complete atrial diagnostics in both VVI and VDD pacing modes.

** for patients with VDD pacing mode.

Device programming differed between the two groups: DX ICD devices were predominantly programmed in VVI pacing mode (87.9%) with a median basic rate of 40 bpm [IQR: 40–40]. In both VVI and VDD pacing modes, DX ICD provides complete atrial diagnostics. All standard dual‐chamber devices were programmed in DDD pacing mode with a basic rate of 60 bpm [IQR: 50–60].

During the first month after implantation, a cumulative low RVP% was found in both groups, but significantly lower in the DX ICD group (0.0% [IQR: 0.0–0.1]) versus the DDD ICD group (1.0% [IQR 0.1–3.8], *p* < 0.001). The median atrial sensing amplitude was significantly higher in the DX ICD group than in the DDD ICD group at 1 month (4.58 mV vs. 3.66 mV, *p* < 0.001) and remained significantly higher throughout the follow‐up until study termination (4.29 mV vs. 3.38 mV, *p* < 0.001) (Table 1).

PS matching identified a subset of 759 patients (396 with DX ICD and 363 with DDD ICD), achieving an absolute standardized mean difference of < 0.1 for all baseline variables (Figure S2). Detailed baseline characteristics of the matched groups are provided in Table S1.

Within the DDD ICD group, 342 patients (42.6%) had a programmed basic rate of < 60 bpm, and 460 patients (57.4%) had a basic rate of ≥ 60 bpm (Table 1). As expected, atrial pacing during the first month was lower when a basic rate was programmed to < 60 bpm (0.2% [IQR: 0.0–3.7]) than to ≥ 60 bpm (13.1% [IQR: 2.2–40.7], *p* < 0.001), while RVP% did not differ between the subgroups.

### 3.2 DDAF Incidence in DX Versus DDD ICD Group

**Figure 2:**
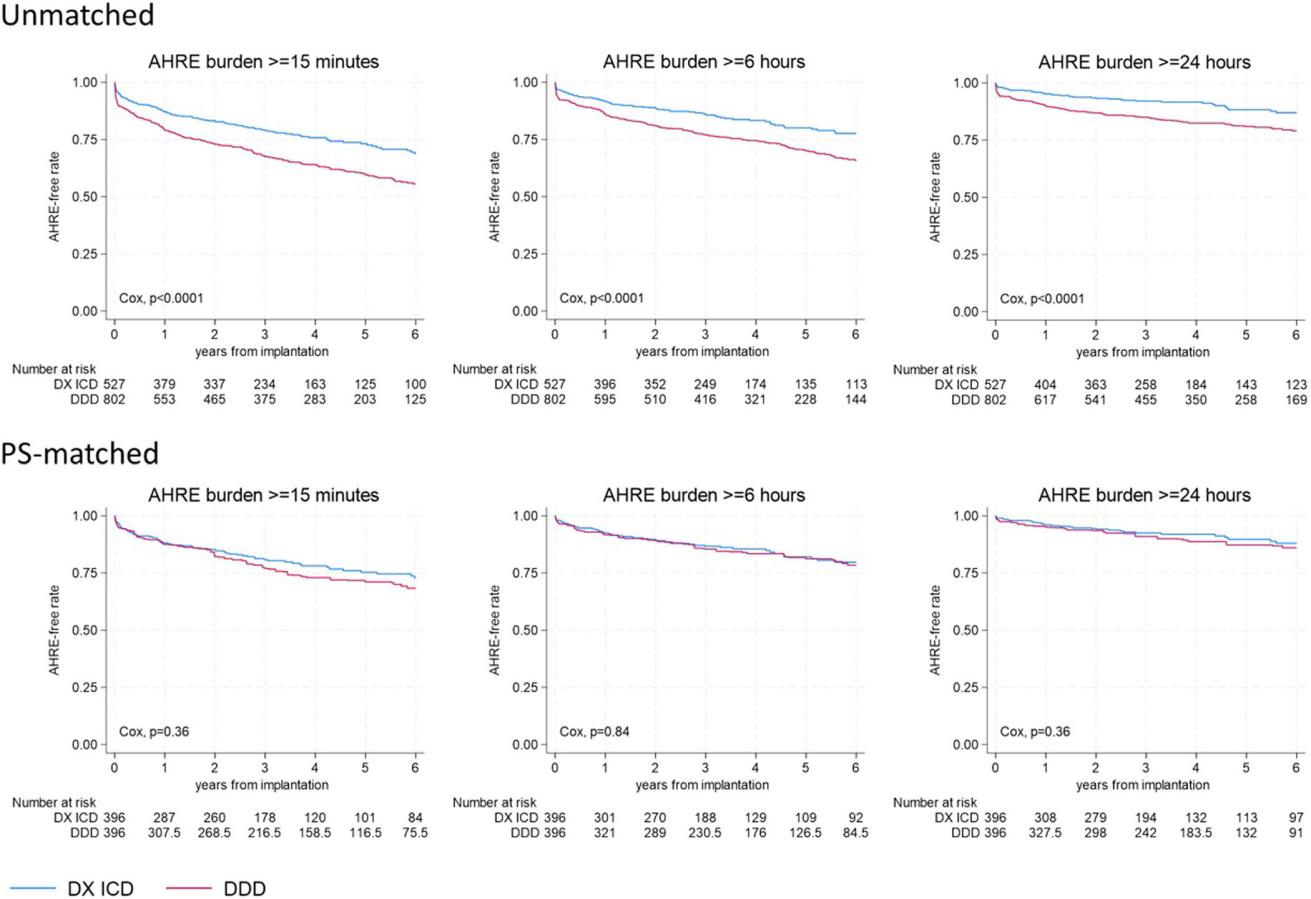
Kaplan–Meier survival curves for device‐detected atrial fibrillation (DDAF) based on different burden cutoffs in DX ICD and DDD ICD groups, analyzed in both the entire cohort (Unmatched) and the propensity score (PS)‐matched cohort.

During a median follow‐up of 4.5 years [IQR: 2.8–6.8], 408 patients (30.7% of all patients) experienced days with DDAF burden ≥ 15 min, 296 patients (22.3%) had days with DDAF burden ≥ 6 h, and 186 patients (14.0%) had DDAF burden ≥ 24 h. The incidence rates of DDAF were significantly lower in the DX ICD group for all burden cutoffs (*p* < 0.0001) in the unmatched cohort. However, when analyzing the PS‐matched cohorts, the incidence rates were similar between groups (Table 2). Thus, the DDAF rates were 6.4 (DX ICD) versus 7.3 (DDD ICD) per 100 patient‐years for the ≥ 15‐min cutoff (*p* = 0.36), 4.3 versus 4.4 for the ≥ 6‐h cutoff (*p* = 0.84), and 2.3 versus 2.8 for the ≥ 24‐h cutoff (*p* = 0.36), respectively. Kaplan–Meier curves reporting DDAF‐free survival for all burden cutoffs, both in unmatched and PS‐matched cohorts, are shown in Figure 2.

**Table 2:** DDAF incidence based on different burden cutoffs in the DX and DDD ICD groups, analyzed in both the entire cohort (unmatched) and the propensity score (PS)‐matched cohort.

DDAF burden cutoff	All patients	DX ICD	DDD ICD	*p* value
≥ 15 min				
Unmatched				
Count (%)	408/1329 (30.7%)	122/527 (23.2%)	286/802 (35.7%)	—
Rate (100‐ppy)	9.9	7.2	11.6	< 0.0001
PS‐matched				
Count (%)	193/759 (25.4%)	83/396 (21.0%)	110/363 (30.3%)	—
Rate (100‐ppy)	6.8	6.4	7.3	0.36
≥ 6 h				
Unmatched				
Count (%)	296/1329 (22.3%)	86/527 (16.3%)	210/802 (26.2%)	—
Rate (100‐ppy)	6.6	4.8	7.8	< 0.0001
PS‐matched				
Count (%)	133/759 (17.5%)	59/396 (14.9%)	74/363 (20.4%)	—
Rate (100‐ppy)	4.3	4.3	4.4	0.84
≥ 24 h				
Unmatched				
Count (%)	186/1329 (14.0%)	47/527 (8.9%)	139/802 (17.3%)	—
Rate (100‐ppy)	3.9	2.5	4.8	< 0.0001
PS‐matched				
Count (%)	83/759 (10.9%)	32/396 (8.1%)	51/363 (14.1%)	—
Rate (100‐ppy)	2.5	2.3	2.8	0.36

Abbreviation: ppy = per‐patient‐year.

### 3.3 DDAF Predictors

Table 3 presents the results of the multivariable analysis for DDAF predictors. Age and RVP% were the only variables significantly associated with DDAF risk in both the entire and PS‐matched cohorts, with no significant effect of device type (DX ICD vs. DDD ICD) or programmed basic rate.

**Table 3:** Multivariable analysis for DDAF predictors in the entire cohort and the propensity score (PS)‐matched cohort.

		Unmatched		PS‐matched	
DDAF burden cutoff	Variable	HR (95% CI)	*p* value	HR (95% CI)	*p* value
≥ 15 min	DX versus DDD ICD	0.73 (0.54–0.98)	0.038	0.94 (0.55–1.61)	0.82
	Age (years)	1.02 (1.01–1.03)	< 0.001	1.02 (1.01–1.04)	0.003
	Sex (female)	0.64 (0.47–0.87)	0.005	0.92 (0.56–1.51)	0.75
	Basic rate (bpm)	1.00 (0.99–1.01)	0.912	1.01 (0.98–1.03)	0.59
	RV pacing (10%)	1.11 (1.06–1.16)	< 0.001	1.15 (1.05–1.26)	0.003
≥ 6 h	DX versus DDD ICD	0.74 (0.52–1.05)	0.09	0.86 (0.45–1.65)	0.65
	Age (years)	1.03 (1.02–1.04)	< 0.001	1.03 (1.01–1.05)	0.003
	Sex (female)	0.61 (0.42–0.88)	0.008	1.00 (0.54–1.84)	0.99
	Basic rate (bpm)	1.00 (0.98–1.01)	0.765	0.99 (0.96–1.02)	0.68
	RV pacing (10%)	1.13 (1.07–1.18)	< 0.001	1.19 (1.08–1.31)	< 0.001
≥ 24 h	DX versus DDD ICD	0.57 (0.36–0.89)	0.013	0.86 (0.45–1.65)	0.65
	Age (years)	1.04 (1.02–1.05)	< 0.001	1.03 (1.01–1.05)	0.003
	Sex (female)	0.54 (0.33–0.89)	0.015	1 (0.54–1.84)	0.99
	Basic rate (bpm)	0.99 (0.97–1.01)	0.40	0.99 (0.96–1.02)	0.68
	RV pacing (10%)	1.11 (1.05–1.18)	0.001	1.19 (1.08–1.31)	< 0.001

Abbreviations: CI = confidence interval, DDAF = device‐detected atrial fibrillation, HR = hazard ratio, RV = right ventricular.

### 3.4 DDAF Incidence by Basic Rate Subgroups in DDD Systems

**Figure 3:**
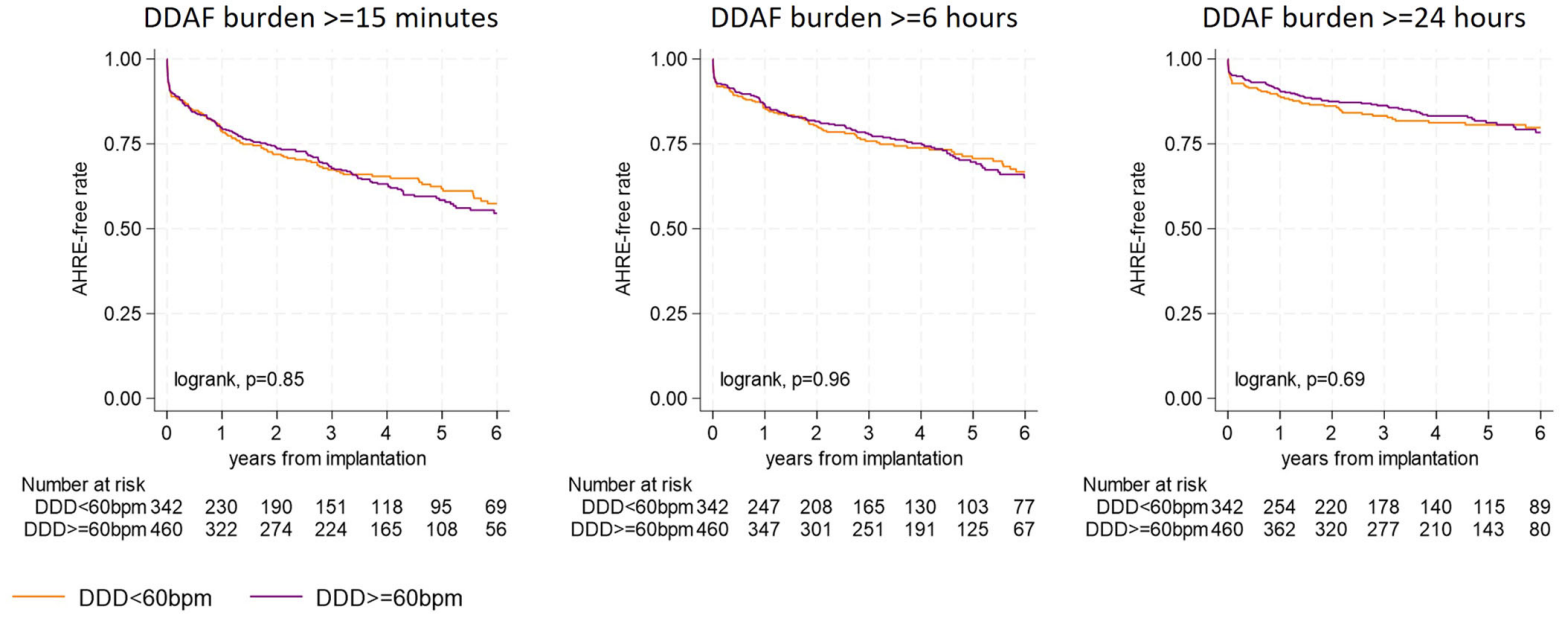
Kaplan–Meier survival curves for device‐detected atrial fibrillation (DDAF) based on different burden cutoffs by basic‐rate subgroups (</≥ 60 bpm) in DDD ICD systems.

Among patients with standard DDD ICDs, no significant differences in study endpoints were observed between those with a programmed basic rate of < 60 bpm and those with a basic rate ≥ 60 bpm (Table S2). The DDAF incidence per 100 patient‐years was 12.0 (< 60 bpm basic rate) and 11.3 (≥ 60 bpm basic rate) for the ≥ 15‐min cutoff (*p* = 0.85), 7.8 and 7.7 for the ≥ 6‐h cutoff (*p* = 0.96), and 4.7 and 5.0 for the ≥ 24‐h cutoff (*p* = 0.69), respectively. Kaplan–Meier curves for DDAF‐free survival by basic rate subgroups in DDD systems are presented in Figure 3.

### 3.5 DX ICD System Upgrades

Ten patients in the DX ICD group (1.9%) underwent device upgrade after a median of 4.9 years [IQR: 2.2–6.8] from initial implantation. Nine of these patients developed an indication for cardiac resynchronization therapy (requiring atrial lead implantation in 6 cases), while one patient received a DDD ICD due to a newly developed indication for atrial pacing.

## 4 Discussion

To the best of our knowledge, this is the first multicenter study with a consistent follow‐up comparing the incidence of new‐onset DDAF in patients implanted with an atrial floating dipole single‐lead ICD and a conventional DDD ICD with a dedicated atrial lead. The main findings of our study are as follows:

1. The DX ICD system detected a burden of DDAF comparable to what is detected by the conventional DDD ICD system.
2. After controlling for baseline confounders using the PS method, RVP% and patient age were the only predictors of DDAF incidence.
3. In a real‐world setting, DDD ICD implantation rates remain high among patients without an indication of atrial pacing. The DX system appeared to be preferred for younger patients or those with secondary prevention indications, congenital cardiomyopathies, or less severe chronic heart failure.

The comparable incidence of DDAF burden in the study groups indirectly confirms the convincing performance of atrial sensing in DX ICD systems. Atrial sensing amplitudes in the DX ICD group were in line with previous reports [12], stable over time, and significantly higher than in the DDD ICD group until the end of the follow‐up period (median 4.5 years). This relatively new finding of our study can be explained by the enhanced atrial input stage amplification in the DX ICD systems.

### 4.1 Atrial Floating Sensor for Subclinical AF Detection

DDAF poses a significant risk for stroke [14] and major adverse cardiovascular events, including heart failure, myocardial infarction, and cardiovascular hospitalization [15, 16]. Recent findings from the NOAH‐AFNET 6 and ARTESiA trials suggest that thromboembolic event prevention in patients with DDAF may be achievable with novel oral anticoagulants, albeit with an increased risk of major bleeding [17, 18]. Early detection of DDAF is important in high‐risk populations, such as ICD recipients, where progression to clinical AF can be rapid. Moreover, atrial arrhythmias have been identified as an independent prognostic factor for increased risk of ventricular arrhythmias and overall mortality [19, 20].

While the atrial sensing floating dipole of the DX ICD system has already shown superior capability in detecting DDAF compared to conventional single‐chamber ICD [9, 21, 22], its performance relative to DDD ICDs has been less thoroughly investigated. Based on a systematic literature review, Pung et al. [21] conducted a meta‐analysis of three non‐randomized studies to compare the likelihood of DDAF detection between DX ICD patients and non‐DX ICD patients, including both VVI and DDD ICDs. However, only a minority of ICDs (15%) were DDD ICDs, precluding a direct comparison between DX ICDs and DDD ICDs. In a prospective study comparing DX ICDs and DDD ICDs, Thomas et al. showed similar DDAF detection capabilities between devices (13% in each group) over a 12‐month follow‐up period [8]. However, the study's reliance on a historical control cohort and significant baseline clinical differences between the groups limited the interpretation of these findings.

In our study cohort, with a longer median follow‐up (4.5 years), the incidence of new‐onset DDAF was higher in the DDD ICD population compared to the DX ICD group across all burden cutoffs (Table 2). However, after balancing the two cohorts for baseline confounders, this difference disappeared for all cutoffs, confirming that the atrial floating dipole of the single‐lead DX ICD system has detection capabilities comparable to a permanent atrial lead. Furthermore, these findings suggest that the absence of an atrial lead with pacing functions or the presence of an atrial dipole in the right atrium does not impact the risk of AF development. Although differences in dipole spacing, structure, and material exist between the DX lead and VDD pacing leads, data from transvenous lead extraction studies of VDD pacing leads suggest that the two annular atrial electrodes often come into contact with the right atrial wall, creating a close interaction at this site and potentially promoting scar formation [23]. This could theoretically lead to conduction slowing, re‐entry, or ectopic activity, thus serving as a non‐pulmonary vein substrate for AF [7, 24]. However, our data provide reassurance in this regard, excluding any proarrhythmic effect of the atrial floating dipole of the DX ICD lead.

### 4.2 Predictors of DDAF Among ICD Populations

DDAF is frequently observed in patients with permanent cardiac implantable electronic devices [25]. Studies have shown that, in patients without a prior diagnosis of clinical AF, the CHADS_2_ and CHA_2_DS_2_‐VASc scores—which account for various comorbidities—are associated with DDAF incidence [25, 26]. However, their predictive accuracy at baseline is limited, as certain components of these scores, such as female sex, have been found to be neutral or even protective [25].

Our PS‐matched multivariable analysis confirmed the well‐established relationship between advanced age and AF [27], demonstrating a 2%–3% increase in DDAF risk per year of age. The analysis also identified the RVP% as the only other significant predictor of DDAF. RVP% showed an estimated hazard ratio ranging from 1.15 to 1.19 for every 10% increase, depending on the DDAF burden cutoff. RVP is known to promote heart failure progression and, particularly in patients with advanced left ventricular dysfunction, can adversely impact left atrial structure and function, theoretically triggering atrial arrhythmias [28]. This detrimental effect of ventricular pacing may be even more pronounced in single‐chamber devices where asynchronous pacing in the VVI mode, with or without retrograde conduction, acts as a strong trigger of AF [29].

Minimizing unnecessary RVP is, therefore, a key goal in ICD programming. Trials such as DAVID II and INTRINSIC RV have demonstrated that outcomes, including AF incidence, are comparable between VVI backup pacing and dual‐chamber modes when AV synchrony is preserved, and unnecessary RVP is limited [30, 31].

In our cohort, DX ICD devices were predominantly programmed in VVI backup mode with a basic rate of 40 bpm (and atrial diagnostics automatically provided), while dual‐chamber devices were programmed in DDD pacing mode, often with an AV hysteresis algorithm. As a result, the overall RVP% was very low (median value, 0.3%). The slightly higher RVP% in the DDD ICD group (median value, 1.0%) could be attributed to the median 13.1% atrial pacing in dual‐chamber devices with a basic rate ≥ 60 bpm, which may prolong intrinsic AV conduction time. In patients without sinus node dysfunction and including a small percentage of patients with baseline AV conduction disturbances (2.8%), DX ICDs provided a potential advantage in terms of straightforward antibradycardia programmability. The option to switch to atrio‐tracking pacing (VDD mode) in case of future AV conduction disturbances enhances this flexibility.

Additionally, in the DDD ICD group, we observed that the programmed basic rate did not affect DDAF incidence. While previous studies have suggested a potential effect of dual‐chamber pacing modality on atrial arrhythmia risk [32, 33], this appears to be relevant primarily in the presence of sinus node dysfunction.

### 4.3 Single‐ and Dual‐Chamber ICDs in Real‐World Practice

Current guidelines recommend single‐chamber ICDs over DDD ICDs for the prevention of sudden cardiac death in patients without the need for atrial or AV sequential pacing [1]. This recommendation is based on the lower risk of device‐related complications associated with single‐chamber systems. However, real‐world practice often diverges from these guidelines. According to data from an American registry, approximately 62% of patients receiving ICDs for primary prevention are implanted with dual‐chamber devices [34]. Of these, 60% lack any pacing indication [34]. Despite evidence linking DDD ICDs to higher in‐hospital and post‐discharge complications, the use of dual‐chamber systems remains common. In our study, 60.3% of patients received a DDD ICD despite lacking an indication for atrial pacing. Previous studies have shown that the addition of an atrial lead is associated with 1.2%–1.3% cumulative incidence of major complications at 6 months [35] and serves as an independent predictor of complications compared with single‐chamber ICDs, including a higher risk of pneumothorax or hemothorax (odds ratio: 1.1; 95% confidence interval: 1.0–1.4) and lead dislodgement (odds ratio: 1.3; 95% confidence interval: 1.1–1.6) [5]. The absence of an atrial lead in DX ICD systems is expected to reduce complication rates compared to DDD ICDs; however, large‐scale studies specifically examining this potential benefit have not yet been conducted.

DX ICD systems were predominantly chosen for younger patients, individuals with secondary prevention indications, those with congenital cardiomyopathies, or patients with less severe chronic heart failure. This preference underscores the perception of DX systems as a safer alternative to DDD ICDs, particularly for patients with longer life expectancies and better quality of life. The single‐lead DX system reduces hardware complexity while retaining the capability to record atrial signals, a feature valuable for supraventricular tachycardia discrimination and rhythm assessment [36].

### 4.4 Study Limitations

This study has certain limitations that should be acknowledged when interpreting the findings. First, the retrospective design inherently introduces the possibility of selection bias and residual confounding, despite the use of PS matching to balance baseline characteristics between groups. Second, the generalizability of the findings may be limited due to the specific population studied. The cohort consisted of patients without an indication for atrial pacing, which narrows the applicability of the results to those who do not require dual‐chamber systems for other clinical reasons. Third, the study did not collect data on the specific positioning of the atrial floating dipole or fluoroscopic images, which could have provided insights into how different positions within the right atrium may affect atrial sensing. However, placement of the atrial dipole in the upper third of the right atrium (normally achieved with the 15 cm dipole spacing from the lead tip) has already been reported to be associated with larger atrial sensing amplitudes and rare ventricular far‐field oversensing during follow‐up [37]. Fourth, our analysis was based on the cumulative 24‐h DDAF burden, which precluded a systematic review of all contributing atrial episodes and differentiation between regular and irregular arrhythmic forms. However, the high positive predictive value of atrial high‐rate episode detection reported for DX ICD systems in the literature supports the validity of our approach. For example, a recent large study of DX ICD patients with electrogram‐based adjudication of DDAF episodes by Hindricks et al. demonstrated a positive predictive value of 97.5% (episodes ≥ 6 min) and 99.7% (episodes ≥ 1 h) being true arrhythmia [12]. These findings suggest a negligible effect of false‐positive episodes ≥ 6 min on atrial arrhythmia detection by DX ICD systems. Moreover, in a similar patient population to ours, 98.7% of true arrhythmia episodes lasting ≥ 6 min were adjudicated as AF [12]. Finally, the study's long follow‐up period is a strength in terms of observing DDAF development but introduces potential variability in clinical management practices over time. Changes in guidelines, device programming strategies, and physician decision‐making during the study period may have influenced the results.

## 5 Conclusions

In real‐world clinical practice, DDD ICDs continue to be frequently used, even in cases where there is no indication of atrial pacing at the time of implantation. The DX ICD, equipped with an atrial dipole, appears to be preferred in patients with a longer life expectancy regardless of a potentially higher risk of ventricular arrhythmias.

After PS matching, our analysis revealed that the incidence of new‐onset DDAF was comparable between DX ICDs and conventional dual‐chamber systems. This finding suggests that the DX ICD's atrial rhythm monitoring capabilities are equivalent to those of conventional DDD ICD systems and that the absence of an atrial lead and pacing function does not increase the risk of developing AF.

RVP%, along with advanced age, emerged as an independent predictor of DDAF. Optimal programming of the pacing mode is essential in patients with ICDs to reduce the risk of new‐onset DDAF.

## Conflicts of Interest

Mauro Biffi has held educational activities and received speaker's bureau on behalf of Boston Scientific, Biotronik, and Medtronic. Daniele Giacopelli and Alessio Gargaro are employees of BIOTRONIK Italia S.p.a.; the remaining authors have no major conflicts of interest to disclose.

## Supporting information

Supporting information.

## Data Availability

The data that support the findings of this study are available from the corresponding author upon reasonable request.
