# Supplementary material for: New‐Onset Device‐Detected Atrial Fibrillation in Patients With Atrial Floating Dipole Implantable Cardioverter‐Defibrillators: A Propensity Score‐Matched Comparison With Conventional Dual‐Chamber Systems
Source: J Cardiovasc Electrophysiol. 2025 Apr 2;36(6):1303–13. doi: 10.1111/jce.16666 (PMC12160691; doi:10.1111/jce.16666)
Supplement: Supplementary file 1 — Supporting information. [file JCE-36-1303-s001.docx]

**Supplementary Material**

This appendix has been provided by the authors to give readers additional information about their work.

Supplement to:

**New-onset device-detected atrial fibrillation in patients with atrial floating dipole implantable cardioverter-defibrillators: A propensity score-matched comparison with conventional dual-chamber systems**

Gianfranco Mitacchione et al.

Contents

[1 Extended results: Common support of propensity score 2](#_Toc192260255)

[2 Extended results: Propensity score matching 3](#_Toc192260256)

[3 Table S1. Extended results: Baseline characteristics of PS-matched cohorts 4](#_Toc192260257)

[4 Table S2. Extended results: DDAF incidence for different burden cutoffs by basic rate subgroups (</≥60 bpm) in DDD ICD systems. 6](#_Toc192260258)

# Extended results: Common support of propensity score

**Figure S1:** Common support of propensity score (PS) between patients with ICD DX (Treated) and standard DDD device (Untreated).

# Extended results: Propensity score matching

**Figure S2:** Absolute mean differences for the propensity score variables before matching and after the matching. A 1:1 match using the nearest-neighbor method with replacement identified a subset of 759 patients, with 396 receiving a DX ICD and 363 receiving a DDD ICD device showing <10% absolute standardized mean difference in all covariates. CMP, cardiomyopathy; HF, heart failure; RVP%, right ventricular pacing percentage.

# Table S1. Extended results: Baseline characteristics of PS-matched cohorts

|  | **Total**  **(n=792)** | **DX ICD**  **(n=396)** | **DDD ICD**  **(n=396)** | **P-value** |
| --- | --- | --- | --- | --- |
| Age (years) | 61 (52-70) | 62 (53-70) | 60 (51-69) | 0.97 |
| Sex (female) |  |  |  | 0.67 |
| NYHA Class |  |  |  |  |
| I | 19 (3.6%) | 17 (6.7%) | 2 (0.717%) |  |
| II | 410 (77.1%) | 186 (73.5%) | 224 (80.3%) |  |
| III | 101 (18.9%) | 49 (19.4%) | 51 (18.5%) |  |
| IV | 2.5 (0.5%) | 1 (0.4%) | 1 (0.5%) |  |
| LVEF (%) | 32 (29-40) | 33 (28-40) | 31 (30-40) | 0.17 |
| CHA_2_DS_2-_VASc score | 2 (1-3) | 2 (1-3) | 2 (1-3) | 0.74 |
| Secondary prevention ICD indication | 245 (30.9%) | 121 (30.6%) | 124 (31.3%) | 0.28 |
| Ischemic CMP | 421 (53.1%) | 220 (55.6%) | 201 (50.6%) | 0.38 |
| Non-ischemic CMP | 193 (24.5%) | 85 (21.6%) | 108 (27.3%) | 0.46 |
| Congenital CMP | 112 (14.1%) | 54 (13.6%) | 58 (14.6%) | 0.07 |
| Diabetes | 153 (19.3%) | 76 (19.2%) | 77 (19.4%) | 0.24 |
| ICKD | 81.5 (10.3%) | 40 (10.1%) | 41 (10.6%) | 0.86 |
| Stroke/TIA | 49 (6.2%) | 28 (7.1%) | 21 (5.4%) | 0.29 |
| AV Block | 17 (2.2%) | 14 (3.5%) | 3 (0.9%) | 0.18 |
| Therapy |  |  |  |  |
| Betablockers | 615 (80.3%) | 307 (79.7%) | 308 (80.8%) | 0.95 |
| Diuretics | 438 (57.5%) | 222 (58.3%) | 216 (56.8%) | 0.16 |
| ACE-inhibitors | 386 (50.4%) | 204 (53.3%) | 182 (47.6%) | 0.54 |
| CCB | 110 (14.5%) | 48 (12.8%) | 61 (16.2%) | 0.28 |
| ARB | 75.5 (10.1%) | 42 (11.2%) | 33 (8.95%) | 0.33 |
| Amiodarone | 85.5 (11.3%) | 44 (11.6%) | 41 (10.9%) | 0.86 |
| Pacing mode |  |  |  |  |
| DDD | - | - | 396 (100%) | - |
| VDD | - | 51 (12.9%) | - | - |
| VVI | - | 345 (87.1%) | - | - |
| Basic rate (bpm) | 50 (40-60) | 40 (40-40) | 60 (50-60) | <0.001 |
| AV delay (ms) | 165 (140-200) | 195 (140-245) | 160 (140-200) |  |
| 1-month AP (%) | 2.0 (0.0-12.9) | - | 2.0 (0.0-12.9) | **-** |
| 1-month RVP (%) | 0.0 (0.0-1.0) | 0.0 (0.0-0.2) | 0.6 (0.0-1.6) | 0.37 |
| P wave amplitude at 1 month (mV) | 4.08 (2.88-5.42) | 4.61 (3.11-5.87) | 3.70 (2.64-4.91) | <0.001 |
| P wave amplitude at study termination (mV) | 3.89 (2.43-5.34) | 4.44 (2.65-6.20) | 3.51 (2.35-4.72) | <0.001 |

Data are median (interquartile range) or count (%).

Abbreviations: ACE, angiotensin-converting enzyme; AP, atrial pacing; ARB, angiotensin receptor blocker; AV, atrioventricular; bpm, beats-per-minute; CCB, calcium channel blocker; CKD, chronic kidney disease; CMP, cardiomyopathy; ICD, implantable cardioverter-defibrillator; LVEF, left ventricular ejection fraction; NYHA, New York Heart Association; PS, propensity score; RVP, right ventricular pacing; TIA, transient ischemic attack.

# Table S2. Extended results: DDAF incidence for different burden cutoffs by basic rate subgroups (</≥60 bpm) in DDD ICD systems.

| **DDAF burden cutoff** | **DDD ICD** | **DDD <60 bpm** | **DDD ≥60 bpm** | **P-value** |
| --- | --- | --- | --- | --- |
| ≥15 minutes |  |  |  |  |
| Count (%) | 286/802 (35.7%) | 121/342 (35.4%) | 165/460 (35.9%) | 0.85 |
| Rate (100-ppy) | 11.7 | 12.0 | 11.3 |  |
| ≥6 hours |  |  |  |  |
| Count (%) | 210/802 (26.2%) | 90/342 (26.3%) | 120/460 (26.1%) | 0.96 |
| Rate (100-ppy) | 7.8 | 7.8 | 7.7 |  |
| ≥24 hours |  |  |  |  |
| Count (%) | 139/802 (17.3%) | 62/342 (18.1%) | 77/460 (16.7%) | 0.69 |
| Rate (100-ppy) | 4.8 | 4.7 | 5.0 |  |

AHRE, atrial high-rate episode; bpm, beats per minute; ppy, per-patient-year.
